# The LAMB3–ITGA6 axis orchestrates epithelial repair in periodontitis via hemidesmosomal regulation and keratinization modulation

**DOI:** 10.3389/fcell.2026.1764896

**Published:** 2026-03-25

**Authors:** Yulong Zhang, Xing Wu, Fei Xu, Qianhui Song, Zhaofen Li, Wenyu Zhen, Rui Wang, Ziwen Sheng, Wansu Sun, Jianguang Xu, Haitao Wang, Hengguo Zhang

**Affiliations:** 1 College and Hospital of Stomatology, Anhui Medical University, Anhui Provincial Key Laboratory of Oral Diseases Research, Hefei, China; 2 Centre for Microvascular Research, William Harvey Research Institute, Queen Mary University of London, London, United Kingdom

**Keywords:** epithelial keratinization, hemidesmosome, ITGA6, LAMB3, periodontitis

## Abstract

**Objective:**

Periodontitis manifests as dysregulated epithelial-stromal interactions and resultant tissue destruction, yet the regulatory mechanisms of the pivotal hemidesmosomes after periodontitis treatment remain elusive.

**Methodology:**

We utilized single-cell sequencing to profile the gene expression pattern of gingival epithelium in periodontitis patients with treatment. By adding retinol (keratinization inhibition) and BMS493 (keratinization promotion) in the human oral keratinocyte, we established and confirmed the *in vitro* cellular keratinization model. The LAMB3-ITGA6 axis expression and interaction were identified in periodontitis gingival tissues and keratinization model. Thereafter, we further validated the regulation of LAMBE via gene knockdown and overexpression process. Finally, we used LAMB3 overexpression lentiviruses to verify the regulatory processes during wound healing in a mouse dorsal full - thickness skin wound model.

**Results:**

Seven distinct epithelial subpopulations were resolved, revealing a bifurcated repair trajectory where one fate selectively activates hemidesmosome-associated genes. In which, the LAMB3–ITGA6 axis emerged as a central hub for coordinating epithelial adhesion and strength. Retinoic acid reciprocally regulated this axis, with its pan-receptor antagonist BMS-493 accelerating epithelial cell keratinization. Importantly, the expressions of cytokeratin family were consistently downregulated in the LAMB3 knockdown group and upregulated in the LAMB3 overexpression group. *In vivo*, mice treated with LAMB3 overexpression lentivirus showed accelerated wound healing and increased cytokeratin 8 expression on dorsal skin.

**Conclusion:**

This study identifying the LAMB3–ITGA6 axis as the target for promoting epithelial repair, which offer a precision medicine framework for periodontal healing.

## 1 Introduction

Periodontitis, a globally prevalent chronic inflammatory disease, progressively destroys teeth-supporting apparatus, such as the periodontal ligament, cementum, and alveolar bone (Villoria et al., 2000; Hajishengallis, 2000). In addition to, the dysregulated interaction between the subgingival microbiome and the host immune response, which disrupts periodontal tissue homeostasis (Lamont et al., 2018; Frencken et al., 2017; Teles et al., 2000). The regeneration of the periodontal epithelium remains comparatively underexplored, particularly in terms of their intrinsic repair mechanisms after therapeutic intervention (Zhou and Graves, 2022; Balta et al., 2021).

Epithelial regeneration and barrier repair are pivotal for periodontal healing (Ba et al., 2021; Susin et al., 2000). This mainly depends on coordinated keratinocyte proliferation, differentiation, and stable adhesion to the underlying basement membrane (BM) (Nanci and Bosshardt, 2000; Groeger and Meyle, 2000). Hemidesmosomes are the special type of junctional complexes that enables epithelial cells be fixed to the BM(12). Meanwhile, the junctional epithelium also attaches tightly to the tooth surface via hemidesmosomes, forming the epithelial–tooth attachment (Dixon et al., 2000). The core mechanism involves the laminin-332 (α3β3γ2 heterotrimer encoded by LAMA3, LAMB3, LAMC2) ligand within the BM binding to the α6β4 integrin receptor (ITGA6/ITGB4) on keratinocytes (Baker et al., 1996; Sugawara et al., 2008). Crucially, the degradation of these adhesive complexes resulted in a loss of attachment to the junctional epithelium (Shimono et al., 2003). Furthermore, although retinoid signaling is known to influence junctional epithelium, its specific impact on the periodontal regenerative niche and its therapeutic reversal potential remain unclear (Eckert and Rorke, 1989; Presland and Dale, 2000).

Here, we employ an integrative translational approach to dissect the molecular network that governs epithelial repair dynamics during periodontitis healing. Single-cell RNA sequencing of human tissues revealed a fate bifurcation in healing epithelia, alongside the dynamic regulation of the hemidesmosome components LAMB3 and ITGA6. We identify a LAMB3-ITGA6 axis governing adhesion and demonstrate that retinoid antagonism enhances keratinocyte adhesion by coordinately upregulating this axis. Functional validation, through genetic manipulation *in vitro* and LAMB3 overexpression *in vivo*, confirmed that targeting this module accelerates wound healing. This work establishes the LAMB3-ITGA6 signaling node as a fundamental coordinator of adhesion-differentiation coupling, presenting its targeted enhancement as a promising therapeutic strategy for periodontal regeneration.

## 2 Materials and methods

### 2.1 Cell culture and treatment

The human oral keratinocyte (HOK) cell line was cultured in DMEM (Gibco, 11330032) supplemented with 10% (v/v) fetal bovine serum (FBS; Gibco, 10437028) and 1% (v/v) penicillin-streptomycin. Cells were maintained at 37 °C in a humidified atmosphere of 5% CO_2_/95% air with media refreshed every 48 h (Gao et al., 2025). HOK cells were tested negative for *mycoplasma* contamination and authenticated via STR profiling (Shanghai Kuisai Biotechnology Co. LTD, Shanghai, China).

HOK cells were seeded in 6-well plates (5 × 10^4^ cells/cm^2^) and cultured at 37 °C/5% CO_2_ until 70%–80% confluency. For keratinization inhibition: cells were treated with 0, 1, or 10 μM all-trans retinoic acid (MCE, HY-14649). For keratinization promotion: cells received 0, 100, or 500 nM BMS 493 (MCE, HY-108529), a pan-retinoic acid receptor antagonist (Yang et al., 2022; Zhou et al., 2023). Drug-containing media were replenished every 48 h to maintain constant concentrations. On day 7 post-treatment, cells were harvested for analysis of markers (e.g., KRT8, KRT18) via Western blotting.

### 2.2 Sample collection

Sample collection was conducted during dental implant surgery and alveolar ridge repair procedures. For health subjects, periodontal tissues were collected from teeth extracted for orthodontic purposes. These individuals exhibited no bleeding on probing, probing depths <3 mm, and no clinical attachment loss or alveolar bone loss. The periodontitis subjects demonstrated active disease markers, including bleeding on probing, probing pocket depths >6 mm, and alveolar bone loss exceeding 60% of the root length. All participants provided written informed consent, and the study was approved by the Ethical Committee Department at the College and Hospital of Stomatology of Anhui Medical University (Approval No. T2021014).

### 2.3 Single-cell RNA sequencing (scRNA-seq)

The raw single-cell RNA sequencing data for this study were sourced from the Gene Expression Omnibus database under accession number GSE171213. In which, periodontal tissue samples of PD patients with treatment (PDT) (n = 3), including alveolar bone, periodontal ligament, and gingival tissues. The subjects showed minimal marginal gingival inflammation but retained advanced clinical attachment loss and bone loss (>60% of root length). Periodontal tissues from PDT subjects were collected from teeth extracted after being judged irrational to treat.

Single-cell transcriptomic data analysis was performed by NovelBio Bio-Pharm Technology Co. Ltd. using the NovelBrain Cloud Analysis Platform. Raw sequencing data were processed with Cell Ranger (v7.1.0) for alignment, barcode assignment, and unique molecular identifier (UMI) counting. Rigorous quality control steps were applied: cells expressing <200 genes, cells with >20% mitochondrial reads, and potential doublets identified via the DoubletFinder algorithm (v2.0.3) were excluded from downstream analyses. Clustering and dimensionality reduction were conducted using Seurat (v4.3.0) with Uniform Manifold Approximation and Projection (UMAP). For pseudotemporal trajectory inference, Monocle 2 (v2.22.0) was employed to model differentiation dynamics. Additionally, epithelial subpopulations were re-clustered using the Mutual Nearest Neighbors (MNN) algorithm to correct batch effects and refine cell-state identification. All computational workflows were executed on the NovelBrain platform to ensure reproducibility and scalability as described previously (Wang Z. et al., 2025; Zhang et al., 2025).

The initial unmapped dataset comprised a total of 23351 raw cells across the samples. Following our strict QC thresholds, we retained a final robust dataset of 10,992 high-quality single cells from the PDT cohort. Within the extracted epithelial and keratinocyte compartment (n = 622 cells), the overall median number of genes detected per cell was 544. Specifically, the median genes per cell across the seven distinct epithelial sub-clusters ranged from 342 (in the Basal_IV cluster) to 4,444.5 (in the highly proliferative Pro_KCs cluster). Detailed median gene counts for each subpopulation are as follows: Basal_I (735), Basal_II (569), Basal_III (461), Basal_IV (342), Basal_V (1,926), Spinous (534), and Pro_KCs (4,444.5).

To determine the statistical significance of genes driving cell fate bifurcation, Branched Expression Analysis Modeling (BEAM) was utilized. Branch-dependent genes were strictly defined using a false discovery rate (FDR)-adjusted *P*-value (*q*-value) <0.05. To visualize the dynamic expression patterns across the trajectory, these identified genes were ranked by their *q*-values in ascending order. Ultimately, the top 1,000 most significant genes were selected and clustered into distinct expression modules to construct the pseudotime trajectory heatmap.

### 2.4 Transfection

LAMB3 knockdown was performed using chemically synthesized siRNAs (21–25 bp duplexes; Tsingke Biotechnology Co., Beijing, China). HOK cells were seeded in 6-well plates at 0.5–2 × 10^5^ cells/well in 1.5 mL antibiotic-free medium and cultured to 60%–80% confluency. Transfection complexes were prepared by mixing siRNA with Lipofectamine™ 2000 (Invitrogen) in Opti-MEM® (Gibco). The mixture (total volume 500 μL) was added dropwise to wells, achieving a final siRNA concentration of 50 nM in 2 mL total volume. After 4–6 h incubation, medium was replaced with fresh complete medium. Cells were harvested 48 h post-transfection for downstream RNA extraction (qPCR) or protein analysis (Western blotting).

For plasmid transfection, LAMB3 overexpression was achieved using the recombinant lentiviral vector GV 358 (GeneChem, China), with the empty vector serving as the control. The lentivirus was produced by GeneChem (China). Packaging plasmids were co-transfected into HEK293T cells. Viral supernatant was collected 48 h post-transfection, filtered through a 0.45 μm membrane filter, and stored at −80 °C for subsequent use. Following infection of HOK cells, successful overexpression of LAMB3 in HOK cells was confirmed by Western blotting 72 h later.

### 2.5 Western blotting

Total protein was extracted from either gingival tissues or cultured HOK cells by lysis in RIPA buffer (P0013, Beyotime Biotechnology) containing protease and phosphatase inhibitors. Protein lysates were resolved on 10% SDS-PAGE gels and electrotransferred onto polyvinylidene difluoride (PVDF) membranes (Merck Millipore). Membranes were blocked with 5% non-fat dry milk, followed by overnight incubation at 4 °C with primary antibodies (see Supplementary Table S1). After rigorous washing, membranes were probed with species-matched horseradish peroxidase (HRP)-conjugated secondary antibodies for 1 h at room temperature. Protein bands were visualized using an enhanced chemiluminescence (ECL) detection kit.

### 2.6 Immunoprecipitation (IP)

For immunoprecipitation, 4–6 × 10^8^ HOK cells were lysed in 1.0 mL ice-cold RIPA buffer (Beyotime) for 30 min. After centrifugation at 13,000 rpm for 15 min at 4 °C, 50 μL of the supernatant was reserved as the input control. The remaining supernatant was incubated with either 10 μL ITGA6 antibody or 1.0 μg control IgG at 4 °C for 4 h. Subsequently, 40 μL of Protein A/G PLUS-Agarose (Santa Cruz Biotechnology) was added, and the mixture was rotated on a rocking platform overnight at 4 °C. The beads were then washed three times with wash buffer, and the immunoprecipitated complexes were resuspended in 2× SDS-PAGE loading buffer and heated at 95 °C for 10 min prior to Western blot analysis.

### 2.7 RNA sequencing

Total RNA from HOK cells treated with RA (10 µM), BMS 493 (500 nM), or vehicle control was extracted using TRIzol. Libraries were prepared with the NEBNext Ultra II RNA Library Prep Kit and sequenced on an Illumina HiSeq 4000. Raw reads were aligned to the human genome (GRCh38) using STAR (v2.7.10a), and differential expression analysis was performed with DESeq2 (v1.38.3). Gene Ontology (GO) and Kyoto Encyclopedia of Genes and Genomes (KEGG) enrichment analyses were conducted using clusterProfiler (v4.8.1). In summary, all data were processed as previously described (Wang Z. et al., 2025).

### 2.8 Animals

This study complies with the ARRIVE 2.0 guidelines for reporting animal research. All experimental procedures were approved by the Anhui Medical University Institutional Animal Care and Use Committee (IACUC Approval No.: LLSC20232087). Mice were anesthetized with 10% ketamine at a dose of 75 mg/kg. Dorsal wounding procedures were performed under aseptic conditions following confirmation of surgical anesthesia (loss of pedal reflex).

### 2.9 Skin wounding models

Eight 8 to 10-month-old C57BL/6 mice were randomized into two groups: (1) negative control lentivirus (Lv-NC) and (2) LAMB3-overexpressing lentivirus (Lv-LAMB3). All mice received dorsal full-thickness excisional wounds under isoflurane-induced surgical anesthesia. After dorsal depilation, two 6-mm full-thickness wounds were created along the dorsal midline per mouse using a sterile biopsy punch. Immediately post-wounding, multipoint subcutaneous injections (4 sites/wound) of Lv-LAMB3 or empty vector were administered intraperilesionally, with each wound receiving a single treatment. On postoperative day 9, wound tissues and visceral organs (heart, liver, spleen, lung, kidney) were harvested with 4% paraformaldehyde. Tissues underwent quantitative histomorphometric analysis to assess wound healing progression and systemic biocompatibility of lentiviral treatment.

### 2.10 H&E staining

Mouse dorsal tissue samples were trimmed and flattened, then incubated in a small volume of fixative (4% PFA) for initial fixation (15 min) to avoid creases. Following initial fixation, additional fixative was added to complete the fixation process. Visceral samples were directly immersed in fixative. Tissue sections were stained with hematoxylin and eosin (H&E). Slides were observed and imaged for histomorphometric analysis.

### 2.11 Immunofluorescence

Immunofluorescence analysis was performed on 4% PFA-fixed, paraffin-embedded murine dorsal skin. After standard deparaffinization and antigen retrieval in 10 mM citrate buffer (pH 6.0), sections were blocked with 5% BSA for 1 h. Primary antibodies against KRT8 and LAMB3 were applied overnight at 4 °C. Species-matched Alexa Fluor-conjugated secondary antibodies were incubated for 50 min at RT. Nuclei were counterstained with DAPI (Servicebio, G1012). Quantitative analysis was performed in a semi-automated way using ImageJ software.

### 2.12 Statistical analysis

Statistical analysis was conducted using GraphPad Prism 9.4.0. Data are presented as mean ± standard deviation (SD). All experiments were independently repeated at least three times. Statistical significance between two groups was evaluated using Student’s t-test. Multiple comparisons were analyzed by one-way analysis of variance (ANOVA), with significance levels set at *P < 0.05, **P < 0.01, and ***P < 0.001.

## 3 Results

### 3.1 Single-cell transcriptomic profiling hemidesmosome-driven epithelial repair in PDT

**FIGURE 1:**
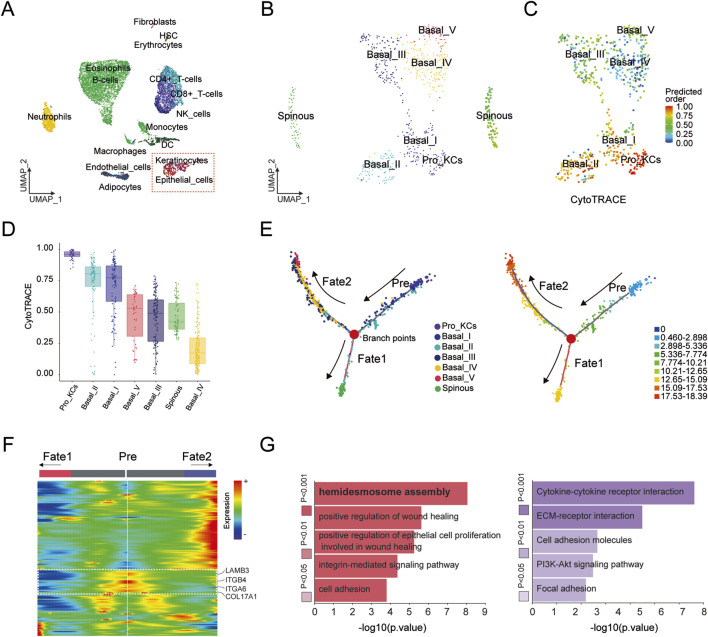
Single-cell transcriptomic profiling hemidesmosome-driven epithelial repair in PDT. **(A)** UMAP projection of 10,992 high-quality single cells from the PDT cohort; the red frame highlights the merged epithelial and keratinocyte compartment. **(B)** Re-clustering of the framed cells in **(A)** resolves seven transcriptionally distinct epithelial subtypes, including basal (I–V), spinous and Proliferation keratinocytes (Pro_KCs). **(C)** CytoTRACE analysis of the seven epithelial subtypes projected onto UMAP reveals a gradient of differentiation potential. **(D)** Box plots of cytoTRACE for the seven epithelial subtypes. **(E)** Pseudotime trajectory inference across epithelial subtypes: left panel shows the branched trajectory embedded in UMAP space; right panel depicts pseudotime progression from progenitor to terminal fates (Fate1, Fate2). **(F)** Heatmap of gene modules (Module 2) at the branch point; pseudotime increases from the central downward (toward Fate1) and upward (toward Fate2). Red bars mark key hemidesmosome-assembly genes (LAMB3, ITGA6, ITGB4, COL17A1) that are selectively activated along the Fate2 trajectory. **(G)** GO and KEGG enrichment of Module 2 identifies positive regulation of wound healing, integrin-mediated signalling, PI3K-Akt pathway and cytokine–cytokine receptor interaction as the most significantly enriched processes (P < 0.01).

To investigate the cellular graph underlying epithelial repair after periodontal therapy, we analysis the PDT single-cell RNA sequencing data from Gene Expression Omnibus database (GSE171213) (Figure 1A). We extracted and re-clustered all epithelial and keratinocyte, yielding seven transcriptionally subpopulations that together map the full depth of the gingival epithelium. Six basal layer clusters (basal I-V) and proliferating keratinocytes (Pro KCs) were identified through KRT5, KRT14, and COL17A1, among which Pro KCs were characterized by elevated CDK1 and MKI67. A seventh cluster expressing KRT1 and KRT10 was assigned to the spinous layer (Spinous) (Figure 1B) (Zhang et al., 2019).

By CytoTRACE analysis, six basal and one spinous cluster along a continuous potency gradient, forecasting robust differentiation capacity in Pro KCs (Figures 1C,D). Downstream of the trajectory’s branching node, pseudotime analysis partitions cells into Fate 1 (barrier-strengthening) and Fate 2 (wound-repair) trajectories (Figure 1E; Supplementary Figure S1A). Heat-map decomposition of branch-point gene modules (module 2) reveals selective activation of hemidesmosome-assembly transcripts—LAMB3, ITGA6, ITGB4 and COL17A1—exclusively within Fate 2 (Figure 1F). GO and KEGG enrichment of module 2 display mechanistically coupling hemidesmosome dynamics to re-epithelialization (Figure 1G). Consequently, hemidesmosome-associated transcripts are prioritized for downstream functional interrogation as putative drivers of repair following periodontal therapy.

### 3.2 LAMB3–ITGA6 axis drives hemidesmosome formation in the wound-reparative epithelial trajectory

**FIGURE 2:**
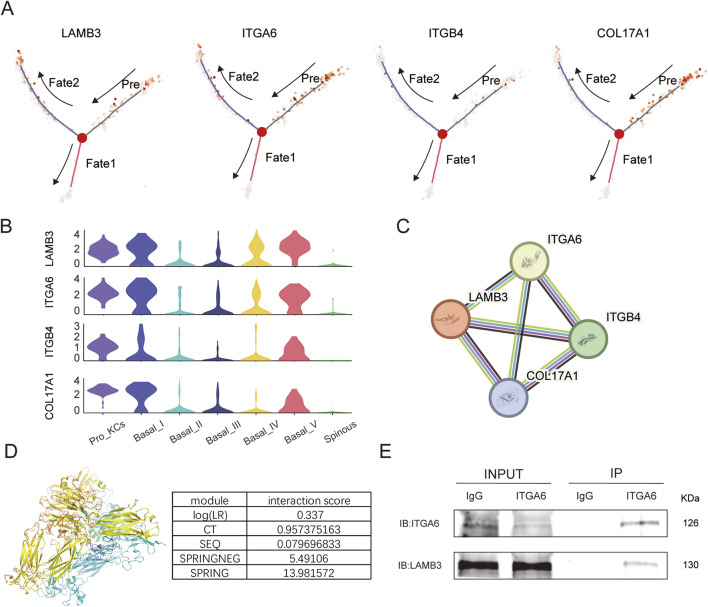
LAMB3–ITGA6 axis drives hemidesmosome formation in the wound-reparative epithelial trajectory. **(A)** Pseudotime trajectory recolored by expression of four hemidesmosome-assembly genes (LAMB3, ITGA6, ITGB4, COL17A1), revealing selective upregulation along Fate2. **(B)** The violin plots illustrate the expression levels of LAMB3, ITGA6, ITGB4, COL17A1 during the healing process of periodontitis. **(C)** STRING network of the six hemidesmosome genes at high-confidence threshold (≥0.700) showing dense, direct protein–protein interactions. **(D)** PEPPI analysis results showing the predicted dimer structure of ITGA6 and LAMB3 (model 1) with a SPRING score of 13.982, overlaid with the dimeric template structure 3FCS (red chain A, blue chain B for model; yellow chain A, cyan chain B for template). The interaction scores of each independent module in PEPPI are shown in the table below. **(E)** Co-immunoprecipitation from HOK cells demonstrating ITGA6-dependent pull-down of endogenous LAMB3.

Pseudotime trajectory mapping confirms selective activation of the four core hemidesmosomal genes (LAMB3, ITGA6, ITGB4, COL17A1) within Fate 2; however, ITGB4 induction remains comparatively muted (Figure 2A). The violin plot shows the expression of these genes in different clusters (Figure 2B; Supplementary Figure S2A). Although both integrin subunits participate in the broader hemidesmosome protein interaction network (Figure 2C), this striking difference in transcriptomic responsiveness highlights ITGA6 as the more dynamically regulated receptor subunit during early repair. Given that the laminin-332 β3 chain—encoded by LAMB3—is indispensable for complex stability, we postulate that ITGA6 and LAMB3 jointly orchestrate hemidesmosome assembly, cell adhesion and subsequent wound healing (Schneider et al., 2007). The Extraction of Predicted Protein-protein Interactions (PEPPI) predicts that the ITGA6-LAMB3 heterodimer superimposes almost perfectly onto the crystallographic 3FCS template (Figure 2D). To complement our structural predictions, endogenous immunoprecipitation assays confirmed that ITGA6 strongly associates and co-precipitates with LAMB3 in HOK cells (Figure 2E; Supplementary Figure S3A). Together with the PEPPI modeling, this supports a tightly coupled interaction between these subunits within the hemidesmosomal complex.

### 3.3 ITGA6–LAMB3 axis in keratinization model of human oral keratinocytes

**FIGURE 3:**
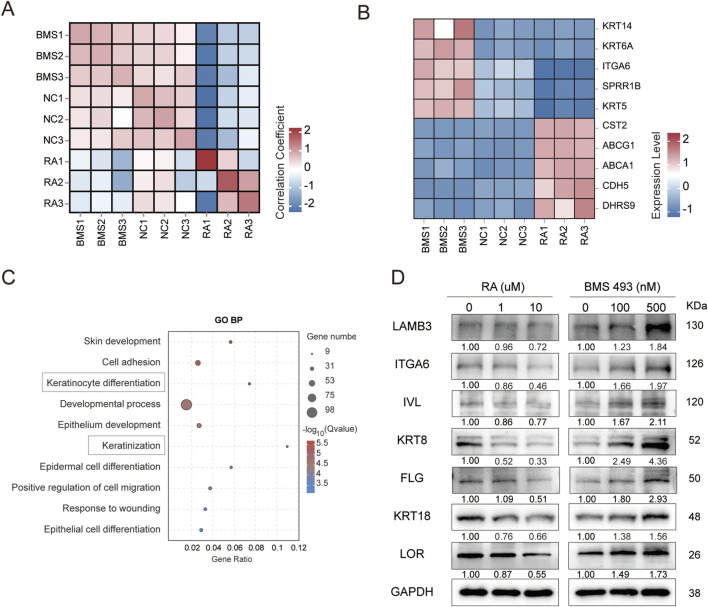
LAMB3–ITGA6 axis in keratinization model of human oral keratinocytes. **(A)** Sample-to-sample Pearson correlation heat map of RNA-seq profiles (n = 3 per condition) derived from HOK cells exposed to vehicle (0.1% DMSO; NC), 10 μM RA, or 500 nM BMS-493. **(B)** Z-score-normalised expression heat map of differentially expressed genes (DEGs) across NC, RA and BMS-493 treatments. **(C)** GO enrichment analysis of DEGs (RA vs. BMS-493) highlighting upregulation of skin/epidermal development, keratinocyte differentiation and cell adhesion. **(D)** Immunoblots of HOK cells treated for 7 days with all-trans retinoic acid (RA; 0, 1, 10 µM) or the pan-RAR antagonist BMS-493 (0, 100, 500 nM) showing dose-dependent modulation of LAMB3, ITGA6, IVL, KRT8, FLG, KRT18, LOR and GAPDH.

HOK Cells treated with retinoic acid for establishing keratinization inhibition model, while cells received BMS 493 for keratinization promotion. We performed RNA sequencing (RNA-seq) analysis on three groups: the RA group, BMS 493-treated group, and control group (Figures 3A; Supplementary Figures S4A,B). The results revealed significant differences in ITGA6 expression levels across the three groups. Specifically, compared to the control group and the RA group, ITGA6 expression was significantly upregulated in samples treated with BMS 493 (Figure 3B).

Additionally, signature marker genes of basal cells, including KRT5, showed significantly higher expression levels in the BMS 493-treated group (Figure 3B). These findings indicate that BMS 493 is effective in promoting epithelial cell proliferation. Notably, keratinization-related marker genes, such as SPRR1B, were also significantly upregulated in the BMS 493-treated group. GO enrichment analysis of differentially expressed genes revealed significant enrichment of terms related to epithelial differentiation and cell adhesion (Figure 3C). This suggests that BMS 493 not only promotes cell proliferation but may also play a role in regulating epithelial cell differentiation.

To dissect the functional synergy between LAMB3 and ITGA6 during epithelial healing, we established an *in vitro* wound model in which HOK cells were exposed to graded concentrations of all-trans retinoic acid (RA; 0, 1, or 10 μM) to mimic healing suppression, or to the pan-retinoic-acid-receptor antagonist BMS493 (0, 100, or 500 nM) to emulate healing promotion. Western blotting revealed dose-dependent regulation: increasing RA lowered LAMB3 and ITGA6 levels, whereas rising BMS493 concentrations markedly raised them. As principal constituents of intermediate filaments, KRT8 and KRT18 endow epithelial cells with mechanical strength and resilience, enabling them to withstand external forces while preserving cellular architecture and epithelial integrity; these proteins mirror the same expression trajectory observed for ITGA6 and LAMB3 (Figure 3D) (Kinumatsu et al., 2009).

### 3.4 The expression pattern of LAMB3–ITGA6 axis in patients with periodontal disease

**FIGURE 4:**
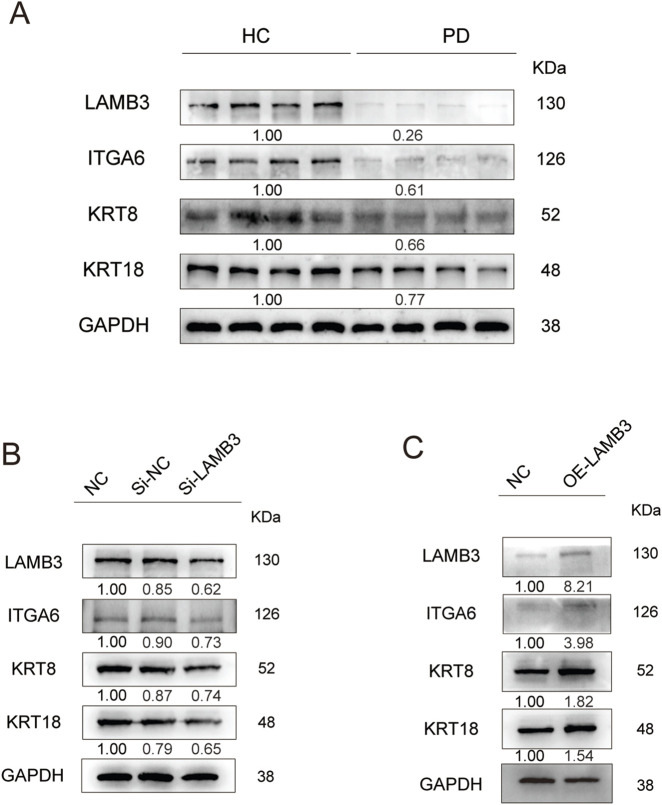
The expression pattern of LAMB3–ITGA6 axis in patients with periodontal disease. **(A)** Western blot analysis showed that compared with the healthy control group, the expression levels of keratin, LAMB3 and ITGA6 in the gingival epithelial tissue of patients with periodontitis were significantly decreased. **(B)** Knockdown of LAMB3 markedly reduces ITGA6 and concurrently downregulates KRT8 and KRT18. **(C)** LAMB3 over-expression elevates ITGA6 while augmenting KRT8 and KRT18 levels.

To translate our mechanistic findings into a clinically relevant framework, we collected gingival tissue from patients with periodontitis alongside age-matched, periodontally healthy controls. Immunoblotting revealed that ITGA6, LAMB3, KRT8, and KRT18 were significantly downregulated in diseased tissue compared with healthy tissue, corroborating the inhibition observed in our *in vitro* wound-healing model (Figure 4A).

Given the coordinated upregulation of LAMB3 and ITGA6 in BMS 493-treated HOK cells, we hypothesized that LAMB3-mediated extracellular signaling might transcriptionally regulate both adhesion (ITGA6) and strength (KRT8/18) programs. To test this, we performed gain- and loss-of-function experiments: 1) siRNA-mediated LAMB3 knockdown markedly reduced ITGA6, KRT8, and KRT18 expression (Figure 4B; Supplementary Figure S5A). 2) lentiviral LAMB3 over-expression significantly elevated their levels (Figure 4C; Supplementary Figure S5B). These results position LAMB3 as the prominent modulator of the adhesion-differentiation axis in keratinocytes, suggesting that epithelial remodeling is mechanistically linked to LAMB3-driven transcriptional activation.

### 3.5 LAMB3 promotes epithelial healing of the skin wound in mice

**FIGURE 5:**
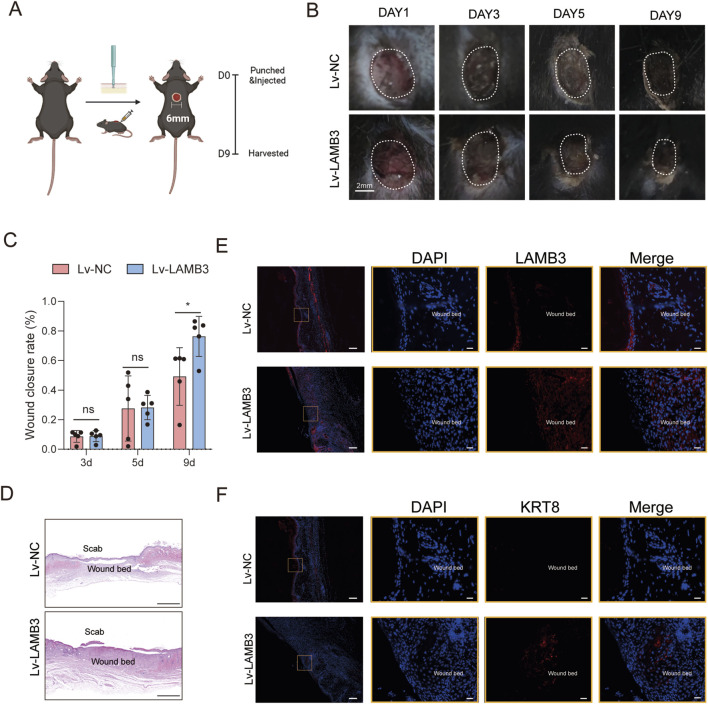
LAMB3 promotes epithelial healing of the skin wound in mice **(A)** Schematic of the full-thickness excisional wound created on the mouse dorsum. **(B)** Accelerated wound closure kinetics with Lv-LAMB3 therapy. Scale = 2 mm. **(C)** Wound closure rate (n = 5). **(D)** Enhanced tissue reorganization in Lv-LAMB3 wounds (H&E, day 9). Scale = 500 μm. **(E)** Immunofluorescence confirms intensified LAMB3 deposition in suprabasal layers in Lv-LAMB3 group. Scale bars: 200 μm (overview) and 20 μm (magnified view). **(F)** KRT8 immunofluorescence demonstrates robust increased expression throughout the Lv-LAMB3 neo-epidermis. Scale bars: 200 μm (overview) and 20 μm (magnified view).

Circular full-thickness wounds were made on the mouse dorsum and LAMB3-overexpressing lentivirus (Lv-LAMB3) or empty control lentivirus (Lv-NC) was injected perilesionally (Figure 5A). Wound closure was markedly accelerated in Lv-LAMB3 mice. Histological analysis of day 9 wound beds displayed better healing and denser tissue in Lv-LAMB3 group (Figure 5D), and immunofluorescence analysis of paraffin-embedded sections demonstrated significantly enhanced expression of LAMB3 and KRT8 in Lv-LAMB3 wounds versus Lv-NC controls (Figures 5E,F). Serial wound imaging at days 0, 3, 5, and 9 post-wounding demonstrated significantly accelerated closure kinetics in Lv-LAMB3 mice versus Lv-NC controls (Figures 5B,C), indicating LAMB3 markedly enhances tissue regenerative capacity.

Simultaneously, histopathological evaluation of H&E-stained visceral organs (heart, liver, spleen, lung, and kidney) demonstrated no adverse effects from lentiviral LAMB3 delivery (Supplementary Figure S6A).

## 4 Discussion

Our study reveals a hemidesmosome-centric transcriptional program, orchestrated by the LAMB3-ITGA6-KRT8/18 axis, that drives gingival epithelial repair after periodontal therapy. This work is of great significance as it provides a single-cell resolution map of human gingiva after treatment. The pseudo-time bifurcation was linked to *in vitro* and *in vivo* functional verification, and it was determined that LAMB3 is a key regulatory factor for keratinocyte adhesion and cellular mechanical strength. Previous studies on LAMB3 and ITGA6 have largely focused on cancer (Yu et al., 2024; Zhang et al., 2023; Gambelli et al., 2024) and their role in junctional epidermolysis bullosa (Schumann et al., 2013; Wang Y. et al., 2025), their functions in epithelial repair have received limited investigation. Here, we performed scRNA-seq on PDT and specifically pinpointed LAMB3 and ITGA6 within the hemidesmosome complex. We then established a bidirectional keratinization gradient using RA and BMS-493 to recapitulate the clinical loss-of-adhesion to re-adhesion trajectory. This method is superior to the traditional single-condition siRNA or overexpression design. Importantly, we verified the existence of this adhesion-loss phase in human periodontal disease. Finally, these findings translated to the mouse model by delivering LAMB3-overexpressing lentivirus into full-thickness dorsal skin wounds. The treatment reproducibly accelerated wound closure without detectable visceral toxicity. Although KRT8 is typically absent in homeostatic adult epidermis, its transient induction in our model likely reflects a wound-induced, highly migratory state rather than aberrant terminal differentiation. This confirms that it is effective both *in vivo* and *in vitro*. Our pseudotime maps and interactome reveal that ITGA6 unexpectedly dominates the repair trajectory, evidenced by its superior network connectivity and functional synergy with LAMB3.

Laminin-332 is a heterotrimer composed of the LAMA3, LAMB3 and LAMC2 chains (Borradori et al., 1999). Early studies focused primarily on the interaction between the α3 chain (LAMA3) and α6β4 integrin, which is essential for epidermal cell adhesion and keratinocyte migration (Jones et al., 1998). However, the three chains are not functionally equivalent. First, LAMB3 is indispensable for the initial assembly and stabilization of the laminin-332 complex (Cheng et al., 1997). Second, in hereditary junctional epidermolysis bullosa, the majority of pathogenic mutations occur in LAMB3, leading to defective assembly and secretion of laminin-332 (Schneider et al., 2007; Muhle et al., 2005). Meanwhile, LAMB3 is an indispensable ligand in the adhesion, differentiation, migration and anti-apoptotic properties of various cell types, including malignant cells. This observation strongly corroborates our finding that LAMB3 acts as the main regulator of the LAMB3-ITGA6 axis governing epithelial keratinization and wound healing.

While our *in vivo* results robustly demonstrate the regenerative capacity of LAMB3, we acknowledge a limitation regarding the tissue model employed. In this study, we utilized a mouse dorsal full-thickness skin wound model as a highly standardized, proof-of-concept system to validate the fundamental biological function of the LAMB3-ITGA6 axis in driving epithelial migration and barrier restoration. The core molecular mechanism of hemidesmosome assembly is highly conserved between skin and oral mucosa (Borradori et al., 1999; Presland and Dale, 2000); however, these tissues possess distinct embryonic origins, local microbiomes, mechanical stresses, and healing kinetics, with oral mucosal wounds uniquely characterized by rapid, rapid repair (Overmiller et al., 2022). Therefore, the microenvironmental differences between the dorsal skin and the periodontal niche must be considered. Future investigations employing periodontal-specific *in vivo* models—such as standardized palatal gingival excisions or ligature-induced periodontitis models in larger mammals—will be crucial. Such models will account for the complex oral microbiome and salivary factors, ultimately confirming the clinical translational potential of targeting the LAMB3-ITGA6 signaling node specifically for periodontal regeneration.

## 5 Conclusion

Our findings establish the LAMB3-ITGA6 axis as a core driver of periodontal epithelial regeneration, where LAMB3 acts as a master regulator to promote ITGA6-mediated adhesion and direct barrier repair. Targeting this axis accelerates mucosal healing, offering a novel therapeutic target for periodontitis.

## Data Availability

The single-cell RNA-seq data reported in this paper have been deposited in the NCBI Gene Expression Omnibus (GEO) under accession number GSE171213. Additional data are available upon request from the authors.

## Supplementary material

SUPPLEMENTARY FIGURE S1
**(A)** the branch-specific s.score of Pseudotime trajectory inference across epithelial subtypes.

SUPPLEMENTARY FIGURE S2
**(A)** Violin plots illustrate the expression levels of selected members of the integrin family and keratinization markers during the healing process of periodontitis.

SUPPLEMENTARY FIGURE S3
**(A)** Co-immunoprecipitation from HOK cells demonstrating LAMB3-dependent pull-down of endogenous ITGA6.

SUPPLEMENTARY FIGURE S4
**(A)** A visual violin plot of the gene abundance expression of the sample. **(B)** A hierarchical clustering graph of the relationships among all samples.

SUPPLEMENTARY FIGURE S5
**(A)** Validation of LAMB3 knockdown efficiency across siRNA constructs, with si-LAMB3-3 showing maximal suppression. **(B)** Western blot confirming robust LAMB3 over-expression following lentiviral transduction in HOK cells.

SUPPLEMENTARY FIGURE S6
**(A)** Visceral histopathology reveals no treatment-related toxicity.

## References

[B1] Bamashmous S. Kotsakis G. A. Kerns K. A. Leroux B. G. Zenobia C. Chen D. (2021). Human variation in gingival inflammation. Proc. Natl. Acad. Sci. 118 (27), e2012578118. 10.1073/pnas.2012578118 34193520 PMC8271746

[B2] Baker S. E. Hopkinson S. B. Fitchmun M. Andreason G. L. Frasier F. Plopper G. (1996). Laminin-5 and hemidesmosomes: role of the α3 chain subunit in hemidesmosome stability and assembly. J. Cell. Sci. 109 (10), 2509–2520. 10.1242/jcs.109.10.2509 8923212

[B3] Balta M. Papathanasiou E. Blix I. Van Dyke T. (2021). Host modulation and treatment of periodontal disease. J. Dent. Res. 100 (8), 798–809. 10.1177/0022034521995157 33655803 PMC8261853

[B4] Borradori L. Sonnenberg A. (1999). Structure and function of hemidesmosomes: more than simple adhesion complexes. J. Investigative Dermatology 112 (4), 411–418. 10.1046/j.1523-1747.1999.00546.x 10201522

[B5] Cheng Y. S. Champliaud M. F. Burgeson R. E. Marinkovich M. P. Yurchenco P. D. (1997). Self-assembly of laminin isoforms. J. Biol. Chem. 272 (50), 31525–31532. 10.1074/jbc.272.50.31525 9395489

[B6] Dixon D. R. Bainbridge B. W. Darveau R. P. (2000). Modulation of the innate immune response within the periodontium. Periodontology 35 (1), 53–74. 10.1111/j.0906-6713.2004.003556.x 15107058

[B7] Eckert R. L. Rorke E. A. (1989). Molecular biology of keratinocyte differentiation. Environ. Health Perspectives 80, 109–116. 10.1289/ehp.8980109 2466639 PMC1567608

[B8] Frencken J. E. Sharma P. Stenhouse L. Green D. Laverty D. Dietrich T. (2017). Global epidemiology of dental caries and severe Periodontitis–A comprehensive review. J. Clinical Periodontology. 44, S94–S105. 10.1111/jcpe.12677 28266116

[B9] Gambelli A. Nespolo A. Rampioni Vinciguerra G. L. Pivetta E. Pellarin I. Nicoloso M. S. (2024). Author correction: platinum-Induced upregulation of ITGA6 promotes chemoresistance and spreading in ovarian cancer. EMBO Mol. Med. 16 (8), 1981. 10.1038/s44321-024-00099-x 38997596 PMC11319497

[B10] Gao X. Zhou J. Qiao Y. Lin C. Zhang G. Wu Q. (2025). ATP6V0A4 as a novel prognostic biomarker and potential therapeutic target in oral squamous cell carcinoma. BMC Oral Health 25 (1), 1269. 10.1186/s12903-025-06653-4 40721755 PMC12302803

[B11] Groeger S. E. Meyle J. (2000). Epithelial barrier and oral bacterial infection. Periodontol 69 (1), 46–67. 10.1111/prd.12094 26252401

[B12] Hajishengallis G. (2000). Interconnection of periodontal disease and comorbidities: evidence, mechanisms, and implications. Periodontology 89 (1), 9–18. 10.1111/prd.12430 35244969 PMC9018559

[B13] Jones J. C. Hopkinson S. B. Goldfinger L. E. (1998). Structure and assembly of hemidesmosomes. Bioessays 20 (6), 488–494. 10.1002/(SICI)1521-1878(199806)20:6<488::AID-BIES7>3.0.CO;2-I 9699461

[B14] Kinumatsu T. Hashimoto S. Muramatsu T. Sasaki H. Jung H. S. Yamada S. (2009). Involvement of laminin and integrins in adhesion and migration of junctional epithelium cells. J. Periodontal Research 44 (1), 13–20. 10.1111/j.1600-0765.2007.01036.x 18973537

[B15] Lamont R. J. Koo H. Hajishengallis G. (2018). The oral microbiota: dynamic communities and host interactions. Nat. Reviews Microbiology 16 (12), 745–759. 10.1038/s41579-018-0089-x 30301974 PMC6278837

[B16] Muhle C. Jiang Q. J. Charlesworth A. Bruckner-Tuderman L. Meneguzzi G. Schneider H. (2005). Novel and recurrent mutations in the laminin-5 genes causing lethal junctional epidermolysis bullosa: molecular basis and clinical course of herlitz disease. Hum. Genet. 116 (1-2), 33–42. 10.1007/s00439-004-1210-y 15538630

[B17] Nanci A. Bosshardt D. D. (2000). Structure of periodontal tissues in health and disease. Periodontology 40 (1), 11–28. 10.1111/j.1600-0757.2005.00141.x 16398683

[B18] Overmiller A. M. Sawaya A. P. Hope E. D. Morasso M. I. (2022). Intrinsic networks regulating tissue repair: comparative studies of oral and skin wound healing. Cold Spring Harb. Perspect. Biol. 14 (11), a041244. 10.1101/cshperspect.a041244 36041785 PMC9620853

[B19] Presland R. B. Dale B. A. (2000). Epithelial structural proteins of the skin and oral cavity: function in health and disease. Crit. Rev. Oral Biol. and Med. 11 (4), 383–408. 10.1177/10454411000110040101 11132762

[B20] Schneider H. Mühle C. Pacho F. (2007). Biological function of laminin-5 and pathogenic impact of its deficiency. Eur. Journal Cell Biology 86 (11-12), 701–717. 10.1016/j.ejcb.2006.07.004 17000025

[B21] Schumann H. Kiritsi D. Pigors M. Hausser I. Kohlhase J. Peters J. (2013). Phenotypic spectrum of epidermolysis bullosa associated with alpha6beta4 integrin mutations. Br. J. Dermatol 169 (1), 115–124. 10.1111/bjd.12317 23496044

[B22] Shimono M. Ishikawa T. Enokiya Y. Muramatsu T. Matsuzaka K.-i. Inoue T. (2003). Biological characteristics of the junctional epithelium. Microscopy 52 (6), 627–639. 10.1093/jmicro/52.6.627 14756251

[B23] Sugawara K. Tsuruta D. Ishii M. Jones J. C. Kobayashi H. (2008). Laminin‐332 and‐511 in skin. Exp. Dermatology 17 (6), 473–480. 10.1111/j.1600-0625.2008.00721.x 18474082

[B24] Susin C. Fiorini T. Lee J. De Stefano J. A. Dickinson D. P. Wikesjö U. M. (2000). Wound healing following surgical and regenerative periodontal therapy. Periodontology 68 (1), 83–98. 10.1111/prd.12057 25867981

[B25] Teles F. Collman R. G. Mominkhan D. Wang Y. (2000). Viruses, periodontitis, and comorbidities. Periodontology 89 (1), 190–206. 10.1111/prd.12435 35244970

[B26] Villoria G. E. Fischer R. G. Tinoco E. M. Meyle J. Loos B. G. (2000). Periodontal disease: a systemic condition. Periodontology 96 (1), 7–19. 10.1111/prd.12616 39494478 PMC11579822

[B27] Wang Z. Zhen W. Wang Q. Sun Y. Jin S. Yu S. (2025a). NEAT1 regulates BMSCs aging through disruption of FGF2 nuclear transport. Stem Cell. Res. Ther. 16 (1), 30. 10.1186/s13287-025-04156-1 39876006 PMC11776329

[B28] Wang Y. Hess M. E. Tan Y. Esser P. R. Nystrom A. Boerries M. (2025b). Alterations in the microenvironment of junctional epidermolysis bullosa keratinocytes: a gene expression study. Matrix Biol. 135, 12–23. 10.1016/j.matbio.2024.11.005 39615637

[B29] Yang Z. Yu M. Li X. Tu Y. Wang C. Lei W. (2022). Retinoic acid inhibits the angiogenesis of human embryonic stem cell-derived endothelial cells by activating FBP1-mediated gluconeogenesis. Stem Cell. Res. and Ther. 13 (1), 239. 10.1186/s13287-022-02908-x 35672803 PMC9171939

[B30] Yu F. Zeng G. Yang L. Zhou H. Wang Y. (2024). LAMB3: central role and clinical significance in neoplastic and non-neoplastic diseases. Biomed. Pharmacother. 178, 117233. 10.1016/j.biopha.2024.117233 39111076

[B31] Zhang X. Lan Y. Xu J. Quan F. Zhao E. Deng C. (2019). CellMarker: a manually curated resource of cell markers in human and mouse. Nucleic Acids Research 47 (D1), D721–D728. 10.1093/nar/gky900 30289549 PMC6323899

[B32] Zhang C. Cai Q. Ke J. (2023). Poor prognosis of oral squamous cell carcinoma correlates with ITGA6. Int. Dent. J. 73 (2), 178–185. 10.1016/j.identj.2022.05.010 35820930 PMC10023534

[B33] Zhang H. Wang Z. Liu Z. Li X. Sun W. Zhen W. (2025). Nuclear FGF2 orchestrates phase separation-mediated rDNA chromatin architecture to control BMSCs cell fate. Bone Res. 13 (1), 80. 10.1038/s41413-025-00451-y 40993129 PMC12460815

[B34] Zhou M. Graves D. T. (2022). Impact of the host response and osteoblast lineage cells on periodontal disease. Front. Immunology 13, 998244. 10.3389/fimmu.2022.998244 36304447 PMC9592920

[B35] Zhou S. Chen J. Cao R. (2023). Association between retinol intake and periodontal health in US adults. BMC Oral Health 23 (1), 61. 10.1186/s12903-023-02761-1 36726080 PMC9893551

