# Supplementary figures and images for: The LAMB3–ITGA6 axis orchestrates epithelial repair in periodontitis via hemidesmosomal regulation and keratinization modulation
Source: Front Cell Dev Biol. 2026 Mar 25;14:1764896. doi: 10.3389/fcell.2026.1764896 (PMC13057497; doi:10.3389/fcell.2026.1764896)

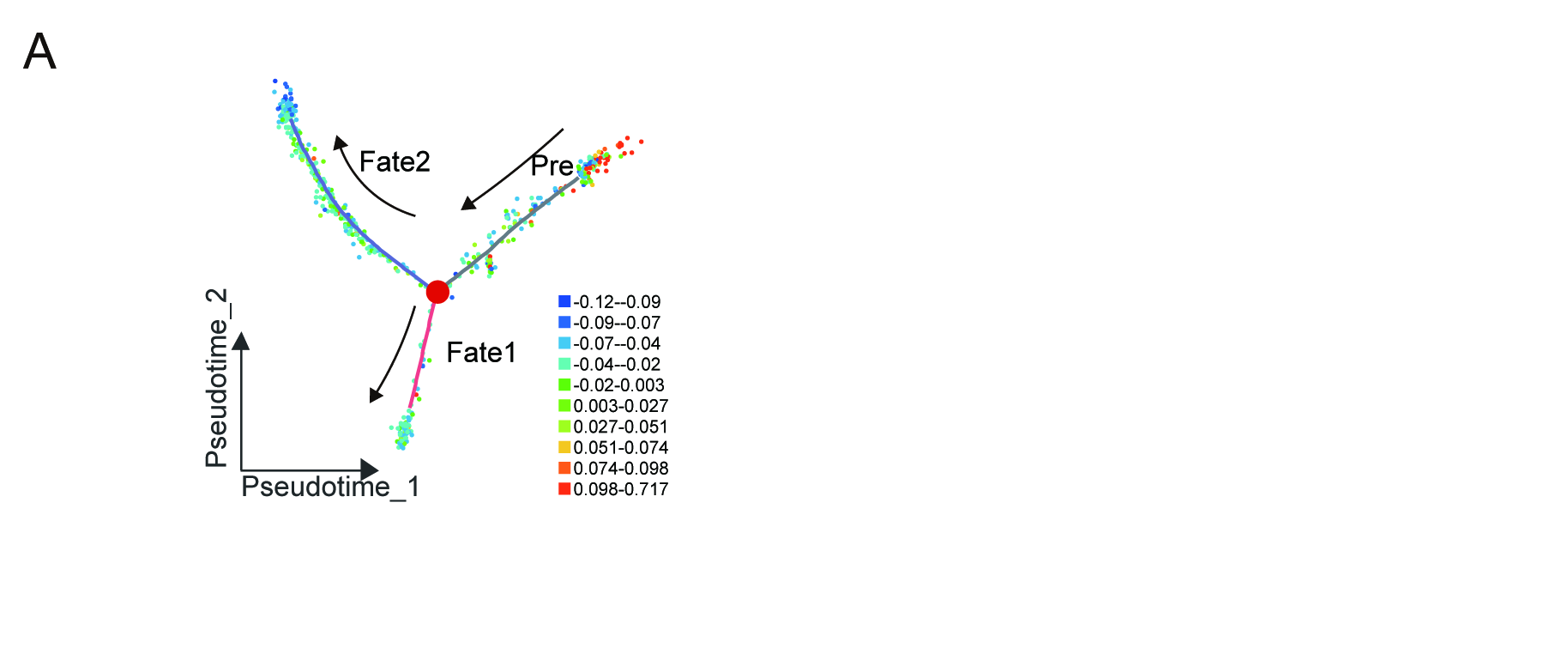

Supplement: Supplementary file 1 [file DataSheet1.zip › FS1.tif]

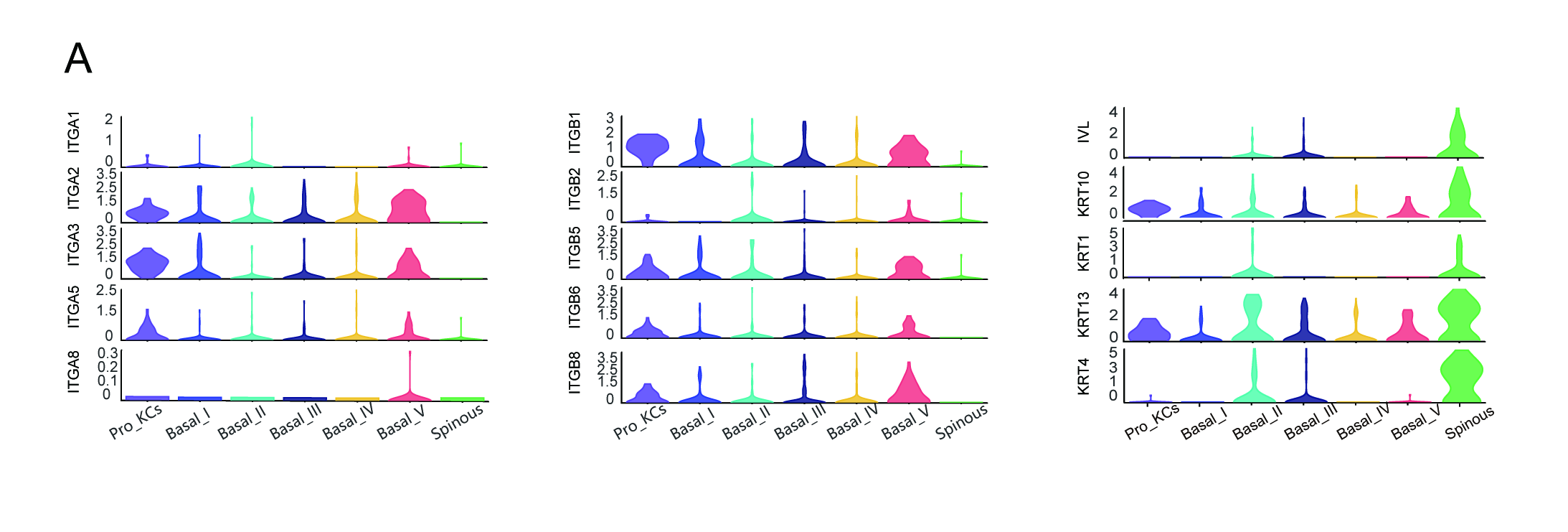

Supplement: Supplementary file 1 [file DataSheet1.zip › FS2.tif]

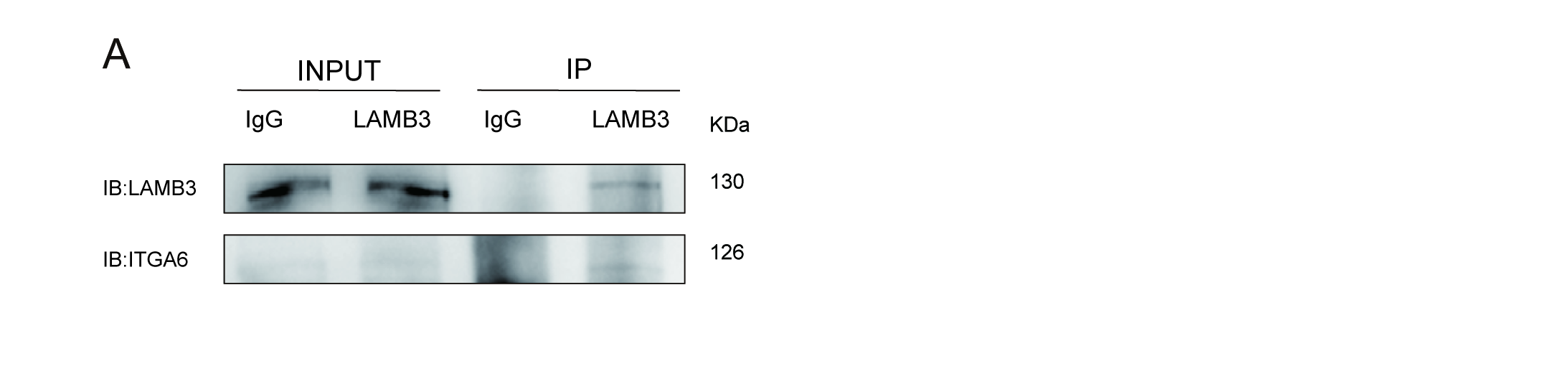

Supplement: Supplementary file 1 [file DataSheet1.zip › FS3.tif]

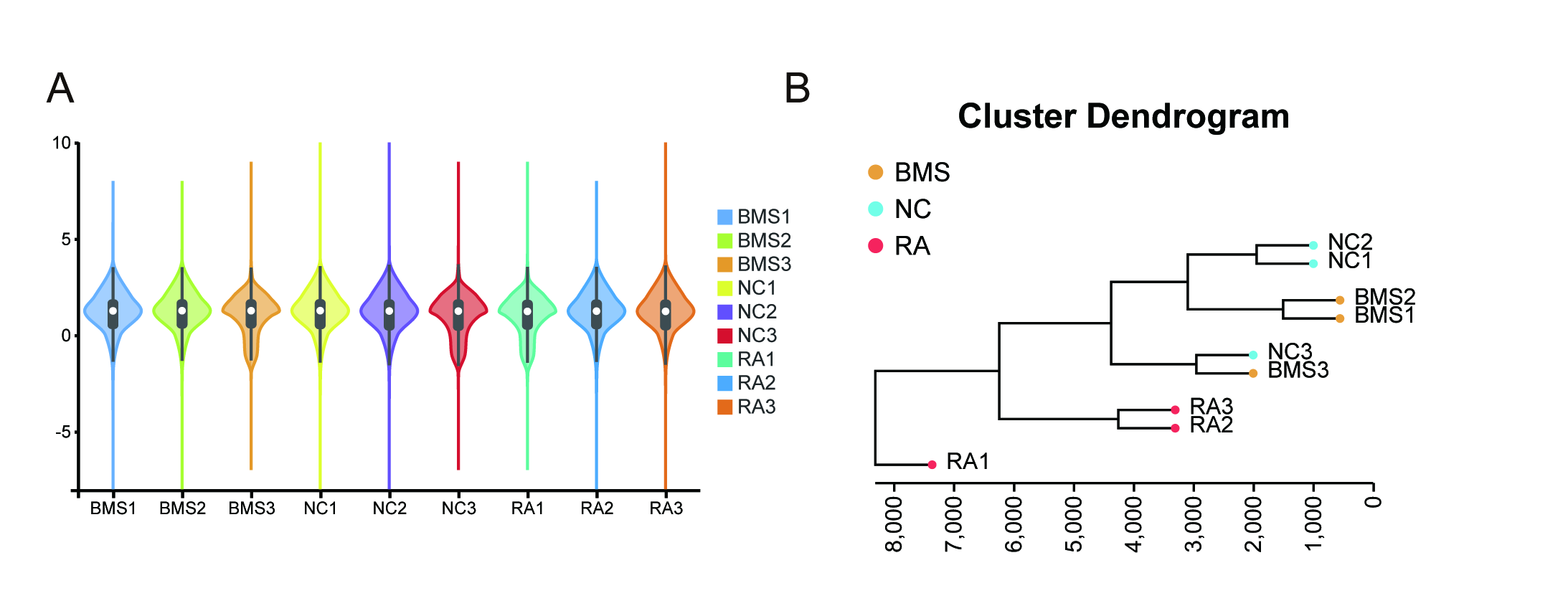

Supplement: Supplementary file 1 [file DataSheet1.zip › FS4.tif]

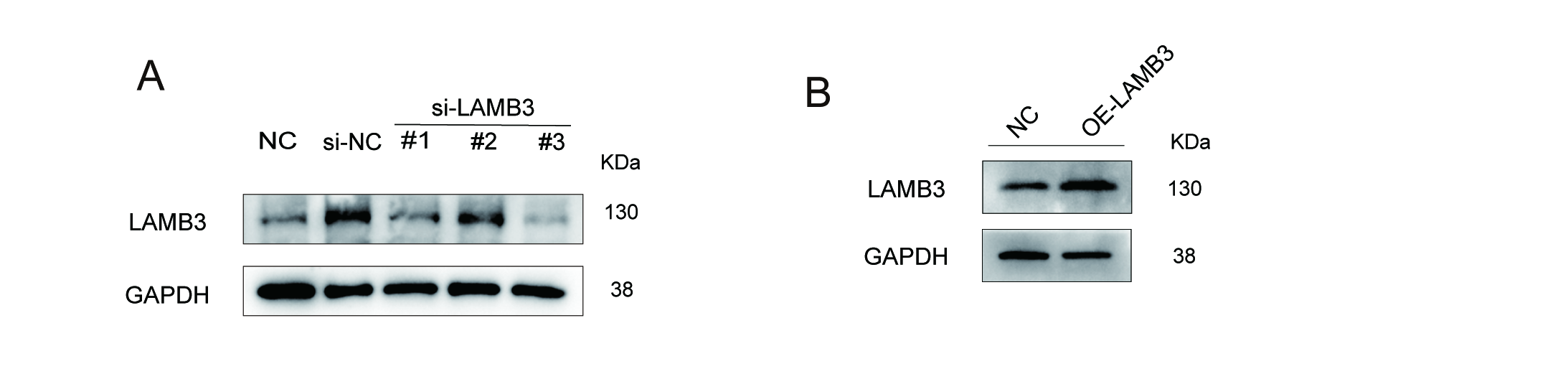

Supplement: Supplementary file 1 [file DataSheet1.zip › FS5.tif]

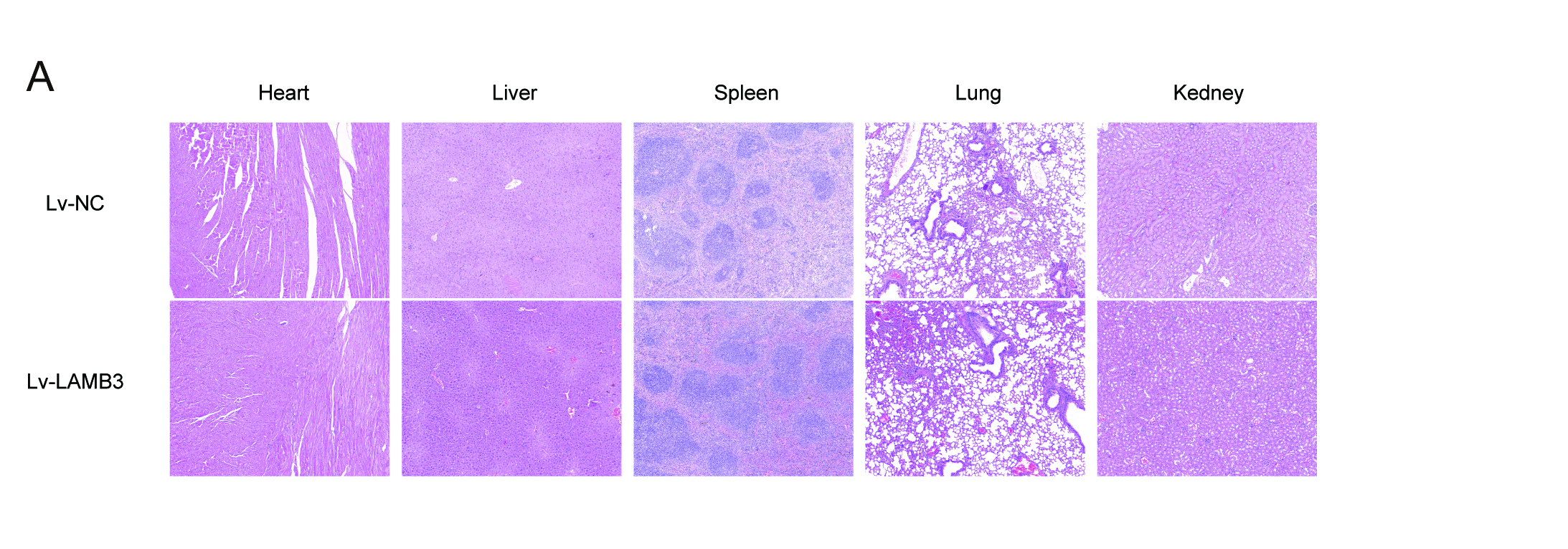

Supplement: Supplementary file 1 [file DataSheet1.zip › FS6.tif]
